# Odontogenic Carcinosarcoma of the Mandible, a Case Report

**DOI:** 10.30476/DENTJODS.2021.91880.1611

**Published:** 2022-09

**Authors:** Asa Rahmat Abadi, Hossein Daneste, Mohammad Ali Ranjbar, DMD, MScD

**Affiliations:** 1 Postgraduate Student, Dept. of Oral and Maxillofacial Pathology, School of Dentistry, Shiraz University of Medical Sciences, Shiraz, Iran; 2 Dept. of Oral and Maxillofacial Surgery, School of Dentistry, Shiraz University of Medical Sciences, Shiraz, Iran; 3 Dept. of Oral and Maxillofacial Pathology, School of Dentistry, Shiraz University of Medical Sciences, Shiraz, Iran

**Keywords:** Odontogenic Carcinosarcoma, Ameloblastic Carcinosarcoma, Malignant Mixed Odontogenic Tumor, Mandible

## Abstract

Odontogenic carcinosarcoma is an extremely rare malignant mixed odontogenic tumor, in which both epithelial and mesenchymal component showing malignant cytology features. Due to paucity of reported cases, clinical appearance is unclear. Present study reports a mandibular odontogenic carcinosarcoma in a 33 years-old male with a history of painless mass in the anterior of mandible. The histopathological examination demonstrated a biphasic malignant neoplasm with both epithelial and mesenchymal component malignant features. There were follicles and strands of odontogenic epithelium, which were lined peripherally by ameloblast-like cells. Mesenchyme of tumor was highly cellular resembling dental papilla. Partial mandibular resection, consisting wide surgical excision with immediate reconstruction was accomplished.

## Introduction

Malignant odontogenic tumors are rare groups of malignant cancers, which arise from remnants of odontogenic epithelium [ 1 ]. One of these tumors is odontogenic carcinosarcoma (OCS), an extremely rare malignant odontogenic tumor in which both the epithelial and the ectomesenchymal components demonstrate malignant features cytologically [ 2 - 6 ].

There are few published case reports of OCS in the literature and they were not specified in WHO classification until 1992 [ 7 ]. Therefore, its clinical appearance is unclear. However the review of literature demonstrates that these rarely malignant cases have exhibited aggressive clinical behavior [ 8 - 9 ].

OCS is related with some tumors which comprise of lesions that range from benign epithelial tumors such as ameloblastoma and ameloblastic fibroma to malignant tumors with metastatic potential like ameloblastic fibrosarcoma [ 9 , 10 ], but due to the scarcity of reported cases, this transformation remains unexplored. There are only twelve OCS cases in the English literature [ 7 - 8 ].

In this report, we describe a case which will be the thirteenth case of OCS arising from an ameloblastic fibroma in the mandible of 33 years-old male patient.

## Case Presentation

A 33-year-old male was presented with a painful mass in the anterior of mandible. Patient suffered from a progressive swelling and alternating paresthesia for approximately 4 months with no complaint of dysphagia, fever, trismus, and weight loss. Past medical history revealed an ameloblastic fibroma in the same region 10 years ago, performed outside our institute, when he was treated by conservative surgery with enucleation. 

**Figure 1:**
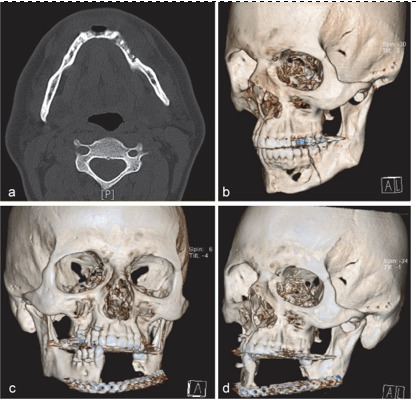
**a:** Computed tomography of odontogenic carcinosarcoma, axial view showing the unilocular lesion extending from the lower right lateral incisor to the up to lower left second premolar, **b:** Three-dimensional image view with buccal perforation and pathological fracture, **c** and **d:** Immediate reconstruction

Physical examination exhibited a poorly defined swelling over the anterior body of mandible with smooth surface roughly 3×4cm in size; no adenopathy was noted. Computed tomography examination displayed a unilocular area of radiolucency with indistinct margins, cortical expansion and buccal cortex perforation (Figure 1). 

**Figure 2:**
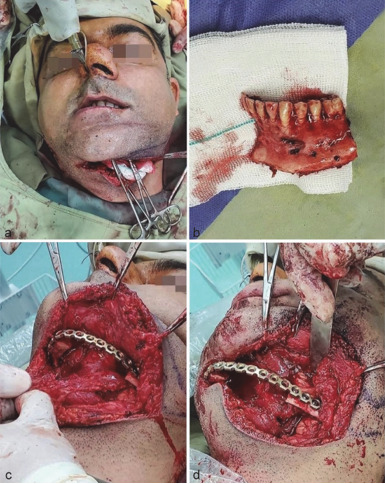
Gross examination; **a:** Partial mandibular resection consisting wide surgical excision, **b:** Cross-section of the resected specimen showing the necrotic fistula on the alveolar ridge, from the lower right lateral incisor up to the lower left first molar, **c** and **d:** Immediate reconstruction

An incisional biopsy was performed. Macroscopically, a whitish soft tissue with elastic texture was observed. According to the clinical imaging and microscopic features, the diagnosis at the time of incisional biopsy was odontogenic carcinosarcoma with malignant characteristic in both odontogenic and mesenchymal parts of tumor. Partial mandibular resection consisting wide surgical excision from the lower right lateral incisor up to the lower first molar with immediate reconstruction was accomplished (Figure 2). 

**Figure 3:**
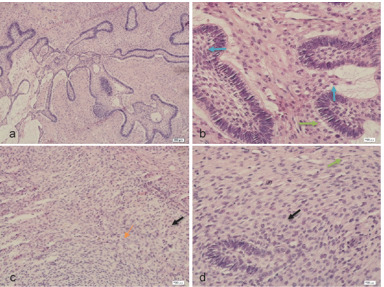
Histopathological examination of the recurred lesion (hematoxylin and eosin stain), **a:** Microscopic image showing odontogenic epithelial follicles formed of ameloblast-like cells on the periphery and stellate-reticulum like cells on the center, which were surrounded by primitive ectomesenchyme resembling dental papilla (100×), **b:** Microscopic image showing hypercellular epithelial follicles with plump hyperchromatic (blue arrow) and pleomorphic cells, bizarre shaped nuclei and increased nuclear/ cytoplasmic ratio (green arrow) (400×), **c:** Microscopic image showing hypercellular ectomesenchyme (100×), **d:** Microscopic image showing atypia in ectomesenchymal component with increased mitosis (black and orange arrows, 200×)

On histopathological examination, all the margins were free of tumor infiltration. Microscopic examination demonstrated a biphasic malignant neoplasm with both epithelial and mesenchymal malignant feature. Epithelial components were in the form of strands and islands with a peripheral palisaded layer of cuboidal or columnar cells and central stellate reticulum like cells. Epithelial component showed malignant features like hyperchromatism of nuclei, pleomorphism, increased nuclear-to-cytoplasmic ratio and abnormal mitotic figures. Mesenchymal element also exhibited malignant features including enlargement of nuclei, hyperchromatism, hypercellularity, and occasional mitoses (Figure 3). The patient is currently being followed up for 16 m-onths with good healing and no sign of recurrence and metastasis.

## Discussion

Malignant odontogenic tumors have exceedingly rare incidence but nonetheless they occur [ 2 ]. Odontogenic malignancies have different origins. Some arise from odontogenic epithelial remnants, residues from embryologic odontogenesis process. Others may develop from preexisting lesions. The mechanism of these transformations is not thoroughly elucidated [ 10 ]. 

As has been demonstrated, the proceeding of odontogenesis involves inductive interaction between the enamel organ and the ectomesenchyme of dental papilla.

It seems that similar induction can cause malignant odontogenic neoplasms like the process occurring in odontogenesis [ 11 ]. 

OCS is a rare malignant mixed odontogenic tumor which both the epithelial and the mesenchymal component present malignant properties. Until now, twelve cases of OCS are reported in the English literature. Four out of twelve cases published in the English literature were considered as de novo [ 10 - 11 ], and other cases were occurred because of previous surgery or were arisen from a preexisting lesion [ 12 ] (Table 1).

**Table 1:** Summary of clinical features of reported cases of odontogenic carcinosarcoma

First Author	Year	Age(yrs.)	Sex	Site	Pre-existing lesion	Follow-up Period(yrs.)	Mortality
Tanaka	1991	63	M	Maxilla	Malignant Ameloblastoma	3.8	Death
Shinoda	1992						
Slater	1999	55	M	Mandible	*De novo*		Survive
Slama	2002	26	F	Mandible	Ameloblastic Fibrosarcoma	3	Death
Kunkel	2004	52	M	Mandible	Ameloblastic Fibrosarcoma	6	Death
DeLair	2007	19	F	Mandible	Ameloblastic Fibroma	2	Survive
Chikosi	2011	9	F	Mandible	Ameloblastoma	2.5	Death
Kim	2013	61	M	Mandible	*De novo*	2	Survive
Santos	2018	42	M	Maxilla	*De novo*	1	Survive
Soares	2019	22	M	Mandible	Ameloblastic Fibrosarcoma		Survive
Soares	2019	19	F	Mandible	*De novo*		Death
Salem	2021	28	M	Mandible	Premature Odontoma	9 months	Survive
Current	2020	33	M	Mandible	Ameloblastic Fibroma	16 months	Survive

Chikosi *et al.* [ 6 ] demonstrated the OCS which has been upraised from ameloblastoma and the OCS that has been reported by DeLair *et al.* [ 5 ] was originated from an ameloblastic fibroma. The cases, which have been reported by Kunkel *et al.* [ 4 ], were developed from ameloblastic fibrosarcoma. 

Although the mechanism of malignant transformation from the benign previous odontogenic lesion is relative unknown, but it is reported that surgical trauma, multiple surgical resection, and radiotherapy seem to have important role in deriving reported cases [ 13 ]. 

In the English literature, there was a male predilection and two cases presented in maxilla [ 14 ]. It is notable that odontogenic carcinosarcoma occurs more commonly in the posterior of mandible, but our case has been existed from anterior part of mandible [ 15 ].

Most of the cases are treated by surgical resection. Some studies revealed that less aggressive resection cause an increase in the possibility of recurrence [ 14 ]. In our case, partial mandibular resection with wide surgical excision was performed and the patient is currently being followed up. 

In the English literature, seven out of the twelve cases showed recurrence of the lesion and only 4 cases showed metastasis.

## Conclusion

This is a case report of odontogenic carcinosarcoma with mixed features of both carcinomatous and sarcomatous components on histopathological evaluation. In spite of limited information about the clinical behavior of OCS, these tumors are very aggressive with high rates of recurrence and metastasis. However, partial resection of mandible seems to be the best treatment, considering the poor outcome of the lesion. 

## Acknowledgement

The authors wish to thank the Research Consultation Center (RCC) of Shiraz University of Medical Sciences for editing this manuscript. 

## Conflict of Interest

The authors declare that they have no conflict of interest.
